# Accurate measurement of endogenous adenosine in human blood

**DOI:** 10.1371/journal.pone.0205707

**Published:** 2018-10-25

**Authors:** Lars Löfgren, Susanne Pehrsson, Gunnar Hägglund, Henrik Tjellström, Sven Nylander

**Affiliations:** 1 Cardiovascular, Renal and Metabolism, IMED Biotech Unit, AstraZeneca, Pepparedsleden 1, Mölndal, Sweden; 2 Q&Q Labs AB, Pepparedsleden 1, Mölndal, Sweden; Tufts University, UNITED STATES

## Abstract

Accurate determination of *in vivo* circulating concentrations of extracellular adenosine in blood samples is challenging due to the rapid formation and rapid clearance of adenosine in blood. A blood collection protocol was developed based on direct sampling of venous blood into, and instant mixing with, a STOP solution developed to conserve *in vivo* adenosine concentrations by completely preventing both its formation and clearance in collected blood. Stable isotope labeled AMP and adenosine spiked into blood *ex vivo* were used in combination with mass spectrometry to evaluate conservation of adenosine and prevention of its formation. A number of approved drugs, including the P2Y_12_ antagonist ticagrelor, have been described to increase extracellular adenosine. This may contribute to its clinical profile, highlighting the importance of accurate measurement of *in vivo* adenosine concentrations.A high sensitive ultra performance liquid chromatography–tandem- mass spectrometry (UPLC-tandem-MS) analytical method for plasma adenosine was developed and validated with a lower limit of quantification of 2 nmol/L. The method demonstrated plasma adenosine stability during sample processing and analytical method performance relevant to human blood samples. The final STOP solution proved able to conserve exogenous adenosine and to prevent adenosine formation from exogenous AMP added *in vitro* to human blood over 15 minutes. The mean endogenous adenosine concentration in plasma prepared from venous blood collected from 10 healthy volunteers was 13 ± 7 nmol/L. Finally, the method was used to demonstrate the previously described concentration-dependent ability of ticagrelor to conserve extracellular adenosine at clinically relevant exposures. In conclusion, we report an optimized sampling protocol and a validated analytical method for accurate measurement of *in vivo* circulating adenosine concentrations in human blood, suitable for use in clinical trials.

## Introduction

**Fig 1:**
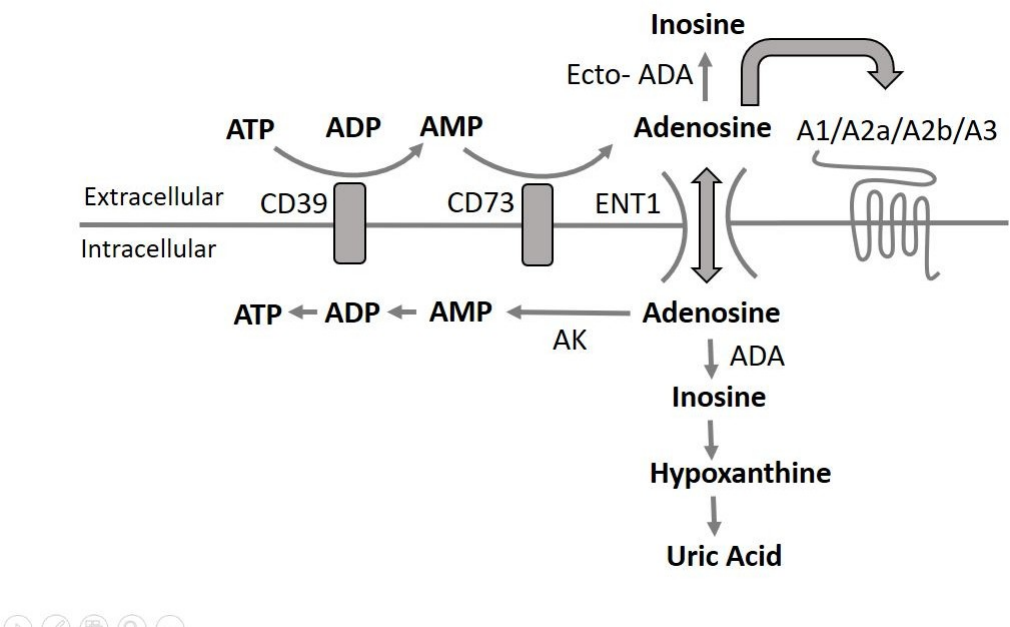
Regulation of blood plasma concentrations of adenosine. The extracellular concentrations of adenosine are depending on the balance between its generation and metabolism. Adenosine is generated by the ecto-5′-nucleotidase CD73 mediated degradation of AMP which is proceeded by the ectonucleoside triphosphate diphosphohydrolase-1 CD39 mediated hydrolysis of ATP and ADP to AMP. The processes regulating adenosine metabolism is more complex and is largely restricted to the intracellular space as adenosine deaminase (ADA) and adenosine kinase (AK) enzyme activity predominantly exist intracellularly [3]. Hence, adenosine transport into cells is a key regulatory step in adenosine metabolism. Cell uptake of adenosine is primarily mediated by the equilibrative nucleoside transporter 1 (ENT1) which acts to keep the adenosine extracellular and intracellular concentration equal. ENT1 expression on red blood cells in the circulation effectively acts as a sink of extracellular adenosine due to the rapid metabolism of adenosine once entering the cells.

Adenosine is an endogenous molecule that has been attributed a number of important physiological effects mediated by its four G-protein coupled receptors (A1, A2a, A2b, A3) including vasodilation, anti-platelet, anti-inflammatory and cardioprotective effects [1]. Its action is largly restricted to its extracellular presence even though intracellular action has been hypothesised [2]. The extracellular concentrations of adenosine depend on the balance between its generation and clearance. Adenosine is formed by the ecto-5′-nucleotidase CD73 mediated degradation of AMP which is proceeded by the ectonucleoside triphosphate diphosphohydrolase-1 CD39 mediated hydrolysis of ATP and ADP to AMP [1]. The processes regulating adenosine clearance are more complex and are likely largely restricted to the intracellular space as adenosine deaminase (ADA) and adenosine kinase (AK) enzyme activity has been described to predominantly exist intracellularly [3]. Hence, adenosine transport into cells is a key regulatory step in adenosine clearance. Cell uptake of adenosine is primarily mediated by the equilibrative nucleoside transporter 1 (ENT1) which acts to keep the adenosine extracellular and intracellular concentrations equal. ENT1 expression on red blood cells in the circulation effectively acts as a sink of extracellular adenosine due to the rapid metabolism of adenosine once entering the cells (Fig 1). Due to the speed of its cellular uptake and of the intracellular metabolism the half-life of circulating adenosine is counted in seconds [4]. Hence the action of adenosine is locally restricted to sites of tissue injury, hypoxia or inflammation where high local concentrations of ATP and ADP will be released whereas the systemic circulating concentrations are generally too low to induce a response. Different publications have reported circulating concentrations at about 10 nmol/L to 1 μmol/L and local concentrations up to 30 μmol/L [1]. There is high variability in the reported concentrations as measuring adenosine concentrations is technically very challenging mainly due to its short half-life in blood [5]. Accurately measuring true *in vivo* adenosine blood concentrations after blood collection requires collecting blood directly into a solution of inhibitors of both adenosine formation and adenosine clearance. As there is no consensus in the use of a specific STOP solution this will contribute to the variability of results reported. Equally important is the speed of blood collection and mixing into the STOP solution. Given that the effects of adenosine can be beneficial in many patients, and that a number of approved drugs have been described to increase extracellular adenosine concentrations, accurate measurement of *in vivo* adenosine concentrations is important. Statins have been described to increase adenosine formation by increasing CD73 activity [6] and the P2Y_12_ antagonist ticagrelor has been described to indirectly inhibit intracellular adenosine metabolism by blocking ENT1 [7–10]. To facilitate the understanding of drug impact on adenosine concentrations we set out to optimize the methodology of blood collection and subsequent analysis. The use of stable isotope (^13^C, ^15^N) labeled adenosine and AMP in combination with mass spectrometry enabled specific and sensitive monitoring of both adenosine clearance and formation and adjustment of the components in the STOP solution. A plasma adenosine UPLC-tandem-MS analytical assay was validated and *in vitro* experiments with ticagrelor confirmed prior data supporting an adenosine mode of action.

## Material and methods

### Chemicals & materials

Adenosine, ticagrelor, dipyridamole, NBMPR (S-(4-nitrobenzyl)-6-thioinosine), EDTA (ethylenediaminetetraacetic acid disodium salt dihydrate), 5-Iodotubericidin (4-Amino-5-iodo-7-(β-D-ribofuranosyl)pyrrolo[2,3-d]pyrimidine), EHNA (erythro-9-amino-β-hexyl-α-methyl-9H-purine-9-ethanol hydrochloride) and PBS (phophate buffered saline, pH 7.4) were from Sigma-Aldrich. AOPCP (α, β-methyleneadenosine 5′-diphosphate) was from Tocris. ^13^C_5_-Adenosine, ^13^C_10_-^15^N_5_-adenosine, ^15^N_5_-AMP, ^15^N_5_-adenosine and ^15^N_4_-hypoxanthine were from Cambridge Isotope Laboratories.

### Preparation of inhibitor solutions

An effective STOP solution needs to block both the formation and clearance of adenosine (Fig 1). The formation of adenosine from AMP (originating from ATP and ADP) via CD73 is inhibited by AOPCP and EDTA. The clearance of adenosine is prevented by a) inhibition of adenosine cell uptake via ENT1 by dipyridamole and NBMPR, b) inhibition of ADA by EHNA and c) inhibition of AK by 5-Iodotubericidin.

Not only a full STOP solution was evaluated but also partial stop solutions to block formation or degradation to demonstrate the role for each process, and hence the need for a full STOP solution. To confirm adequate composition and performance of the final STOP solution it was directly compared with a STOPx2 solution with doubled concentrations of all inhibitors.

The composition of the different inhibitor solutions explored are given in Table 1. PBS (pH 7.4) was used as buffer.

**Table 1:** Composition of inhibitor solutions (μmol/L).

Compound	Target	MIX 1	MIX 2	NO STOP	STOP	STOPx2
EDTA	CD39/CD73	-	15000	-	15000	30000
AOPCP	CD73	-	220	-	220	440
NBMPR	ENT1	100	100	-	100	200
Dipyridamole	ENT1	40	40	-	40	80
5-Iodotubericidin	AK	100	-	-	100	200
EHNA	ADA	100	-	-	100	200

The inhibitor concentration in the final blood:solution mix (2:1) is one third of the concentrations above

Stable isotopes of adenosine (^13^C_5_-adenosine at 1 μmol/L final blood concentration) and AMP (^15^N_5_-AMP at 10 μmol/L final blood concentration) were added to the solutions to explore a) the conservation of exogenous adenosine concentrations over time, b) the inhibition of adenosine formation from exogenous AMP over time, and thereby the relevance of the different components in the inhibitor solution. When comparing the performance of STOP and the STOPx2 solutions ^13^C_1o_-^15^N_5_-adenosine (at 100 nmol/L final blood concentration) was added.

The inhibitor solutions and stable isotopes were prepared at room temperature and stored at -20°C or directly transferred into 2.6 mL S-Monovette K3E tubes (Sarstedt, Nümbrecht, Germany) at a volume of 1.3 mL. Frozen inhibitor solutions were allowed to reach room temperature and were carefully mixed prior to use.

### Blood collection

Blood was collected from healthy Caucasian male and female volunteers by vein puncture of vena cephalica. All subjects abstained from drugs known to affect platelet function for at least 10 days. Informed consent was obtained from all subjects, and the study was performed in accordance with local ethical regulations following approval from the regional ethics committee “Regionala Etikprövningsnämnden i Göteborg”, Sweden (reference number 033–10).

Prior to blood sampling, the membrane screw cap was removed from the S-Monovette tubes and 1.3 mL inhibitor solution was added before resealing the tubes. Immediately prior to blood sampling the tubes were inverted ten times to homogenize the inhibitor solutions. The first about 2 mL of blood was discarded, then blood was collected into the tube with STOP solution, by retracting the piston until it snapped into its stop position, ensuring an end volume of 2.6 mL blood, inducing immediate mixing of the blood with the inhibitor solution in a 2:1 blood:solution ratio. The tubes were gently inverted 5 times to ensure complete mixing. Plasma was prepared at different time points (4, 15 and 30 min) after blood collection by centrifugation at room temperature at 1640xg for 10 min and stored at -20°C until analysis.

### Effects of ticagrelor on adenosine conservation in blood

Ticagrelor in DMSO (final concentrations in blood; 1.0, 3.0, 10 and 30 μmol/L) or vehicle only (DMSO), 2 μL, were pre-incubated with 4 mL whole blood at room temperature for 60 minutes. The pre-incubated blood was then spiked with ^13^C_5_-adenosine in 80 μL PBS to a final blood concentration of 1 μmol/L and mixed by gently inverting the tubes eight times. One minute after the addition of ^13^C_5_-adenosine, 1 mL blood was transferred into 500 μL STOP solution. The blood was centrifuged within 5 minutes at 1640 x g for 10 min at room temperature and plasma was stored at -20°C until analysis.

### Plasma adenosine analysis

The method described is intended for and has been validated for determination of endogenous adenosine in plasma prepared from a blood:STOP solution (2:1) mix. The method is also suitable for assessment of inosine and hypoxanthine but was not validated for these adenosine metabolites.

Stock solutions of 1mmol/L ^13^C_5_-adenosine, for purpose of internal standard and of 3.7 mmol/L adenosine, for purpose of external calibration curve, were prepared in 20% methanol in PBS (pH 7.4).

The external calibration curve for adenosine was prepared by serial dilution of the 3.7 mmol/L stock solution to 500, 166, 56, 19, 6.2 and 2.1 nmol/L in PBS. Internal standard solution, 3333 nmol/L, were prepared from the stock solution in 20% methanol in PBS.

Plasma, 150 μL, and internal standard, ^13^C_5_-adenosine, 33 μl, were first transferred to polypropylene micro tubes and mixed for 10 seconds. Then the mixed samples were added to 10 kDa molecule cut-of spin filter (Amicron ultra 10K, 0.5 mL) and centrifuged at 14000xG for 40 minutes at 4°C. The sample filtrates were collected for subsequent adenosine analysis by UPLC-tandem-MS.

An Agilent 6540 UPLC-tandem-MS instrument operated by the Masshunter software was used. Eight μL plasma sample filtrate or calibration sample was injected. Mobile phase A was 10 mmol/L ammonium formate and 0.1% formic acid (pH 3.2) in milli-Q water and mobile phase B was 10 mmol/L ammonium formate and 0.1% formic acid in 99% methanol. Separation of analytes was achieved on a XSELECT HSS T3 75 x 3 mm, 2.5 μmol/L analytical column using a gradient run from 5% B at 0 min, 17.5% B at 2 min, 90% B at 2.1–3.1 min and 5% B at 3.2–4.6 min with a total run time of 4.6 min. The retention time for adenosine as well as for ^13^C_5_-adenosine was 1.7 min. The mobile phase flow was 0.8 mL/min. The column temperature was 50°C and samples were kept at 20°C.

Endogenous as well as stable isotope labeled adenosine and hypoxanthine were ionized in positive electrospray mode and analyzed by multiple reaction monitoring (Table 2). Dwell times were 50 ms.

**Table 2:** Mass spectrometry settings on Agilent 6540 UPLC-tandem-MS system.

Compound	Transition	Fragmentor	Collision Energy	Accelerator Voltage	Polarity
Adenosine	268.1 > 136.1	118	24	4	POS
^13^C_5_-Adenosine	273.1 > 136.1	118	24	4	POS
^13^C_10_-^15^N_5_-Adenosine	283.1 > 146.1	118	24	4	POS
^15^N_5_-Adenosine	273.1 > 141.1	118	24	4	POS
^15^N_5_-Hypoxanthine	141.1 > 113.1	132	24	4	POS

Endogenous adenosine was quantified using the external calibration curve for adenosine with ^13^C_5_-adenosine as the internal standard according to the bioanalytical validation plan. When ^13^C_10_-^15^N_5_-adenosine was used for investigation of adenosine stability over time, ^13^C_5_-adenosine was used as the internal standard. For the simultaneous investigation of the stability of ^13^C_5_-adenosine and the contribution to adenosine from ^15^N_5_-AMP degradation, ^13^C_10_-^15^N_5_-adenosine was used as the internal standard.

Quantification of endogenous adenosine was performed by the Masshunter software by fitting the observed response factor (area units for the analyte versus the internal standard) for unknown samples to the calibration curve. Quadratic curve fitting was used ignoring origo and using a 1/X weighting factor. The concentration range evaluated was 2.1–500 nmol/L adenosine in plasma samples with a lower limit of quantitation (LLOQ) of 2.1 nmol/L. ^15^N_5_-AMP metabolites were quantified as area units (AU) without the use of a calibration curve. The estimated concentrations (nmol/L) given for ^15^N_5_-adenosine formed from ^15^N_5_-AMP degradation were based on area units and response factors for the analytes and the internal standard respectively. For quantification of ^13^C_5_-adenosine in plasma, the analyte versus internal standard response factors obtained for blood samples were compared to reference samples prepared by replacing blood with PBS when mixing with the inhibitor solution containing exogenous ^13^C_5_-adenosine.

### Validation of the analytical method

The here described analytical method for endogenous adenosine assessment in human plasma was validated according to GCP (good clinical practice) in accordance with a validation plan based on the EMEA Guidelines [11].

Parameters evaluated and validated were selectivity, matrix effects, accuracy, calibration curves, instrument repeatability, carry-over, dilution integrity, lower limit of quantitation (LLOQ) and sample stability. The performed validation tests and results are summarized in S1 Table.

### Effects of perchloric acid extraction versus filtration on plasma adenosine concentrations

Sample preparation for removal of plasma proteins by the filtration method validated here was compared to classical perchloric acid (PCA) extraction followed by neutralization after 5 minutes or after 12 hours at 4°C to test the stability of adenosine and AMP during strong acidic conditions occurring during PCA extraction.

Blood from five healthy subjects was collected into the STOP solution and plasma was prepared 15 minutes after blood sampling. Two aliquots were prepared from each sample. One aliquot was spiked with 10 μmol/L final concentration of AMP to test for adenosine formation in PCA extracts at endogenous and elevated AMP plasma concentrations. All samples contained exogenous ^13^C_10_-^15^N_5_ adenosine (added to the STOP solution prior to blood collection) used to test for adenosine stability in PCA extracts. 300 μL plasma replicates were then prepared.

50 μl 5.0 mol/L PCA was added to each plasma replicate (n = 5). Samples were mixed for 30 seconds and proteins separated by centrifugation at 14000xG for 5 minutes. The pH in the extracts before neutralization was < 1 corresponding to a final PCA concentration of about 700 mmol/L. 200 μl of the protein-free supernatant was neutralized after 5 minutes or after 12 hours by adding 35 μl of 4.0 mol/L potassium hydroxide containing 0.4 mol/L sodium phosphate at pH 7 and 4°C. The KClO_4_ precipitate was removed by centrifugation. 30 μl internal standard (1 μmol/L ^13^C_5_-adenosine) was added to 250 μl of the supernatant prior to analysis of endogenous adenosine and exogenous ^13^C_10_-^15^N_5_ adenosine by UPLC-tandem-MS.

## Results

### Relevance of the different components in the stop solution

**Fig 2:**
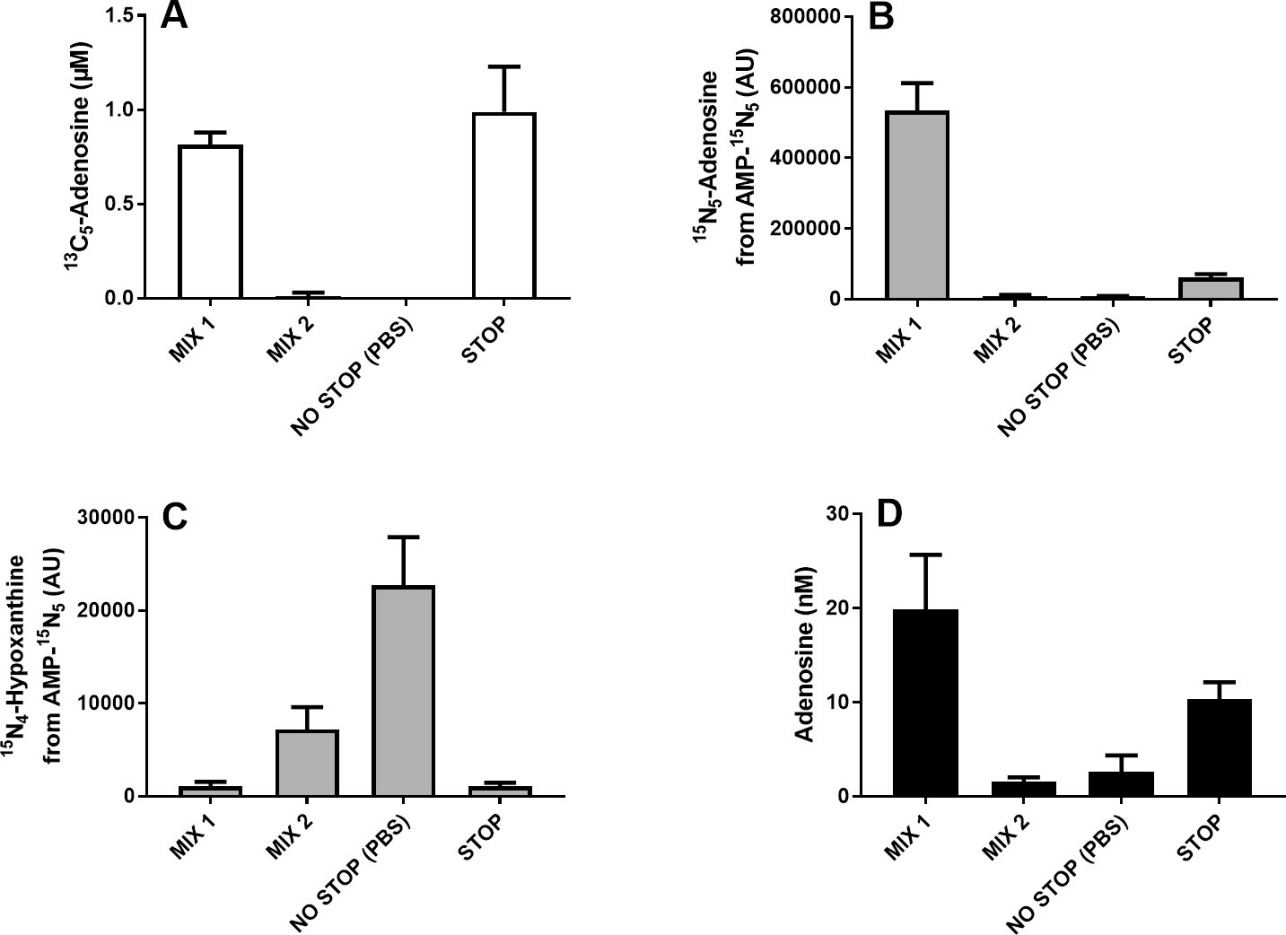
**Effects of inhibitors of adenosine formation and clearance on plasma concentrations of exogenous** ^**13**^**C**_**5**_**-adenosine (A),** ^**15**^**N**_**5**_**-adenosine from exogenous** ^**15**^**N**_**5**_**-AMP (B),** ^**15**^**N**_**5**_**-hypoxanthine from exogenous** ^**15**^**N**_**5**_**-AMP (C), and endogenous adenosine (D).** Venous blood from healthy donors was collected into blood collection tubes pre-filled with inhibitor solutions containing final blood concentrations of 1 μmol/L ^13^C_5_-adenosine and 10 μmol/L ^15^N_5_-AMP. Plasma was prepared within 5 min of blood collection. Data expressed as means ± SD, n = 5.

The impact of adenosine formation and adenosine metabolism was explored by collecting blood into MIX 1 (leaving adenosine formation via CD73 uninhibited) and MIX 2 (leaving adenosine metabolism via AK and ADA uninhibited). The performance of these inhibitor solutions was compared with collecting blood in the complete absence of inhibition—NO STOP—or into the final STOP solution. The measured plasma concentrations of endogenous adenosine, exogenous ^13^C_5_-adenosine and the degradation products from exogenous ^15^N_5_-AMP metabolism (^15^N_5_-adenosine and ^15^N_5_-hypoxanthine) are illustrated in In Fig 2.

Exogenous adenosine was not detectable in samples collected into NO STOP whereas about 100% of added adenosine could be detected in samples collected into STOP solution. Similar to NO STOP, almost no exogenous ^13^C_5_-adenosine could be detected in samples collected into MIX 2 (no AK or ADA inhibition) whereas similar concentrations as in samples collected under STOP conditions could be detected in samples collected into MIX 1 (no CD73 inhibition) (Fig 2A).The concentrations of adenosine formed from exogenous ^15^N_5_-AMP were dramatically higher in samples collected into MIX 1 (no CD73 inhibition) compared to samples collected into STOP. Only trace amounts of adenosine formed from exogenous ^15^N_5_-AMP could be detected in samples collected into MIX 2 (no AK or ADA inhibition) or NO STOP (Fig 2B). Instead the added ^15^N_5_-AMP was found as high amounts of hypoxanthine whereas only trace amounts of hypoxanthine generated from ^15^N_5_-AMP could be detected in samples collected into STOP and MIX 1, both solutions inhibiting adenosine uptake and metabolism (Fig 2C). The highest concentration of endogenous adenosine was observed in samples collected into MIX 1 (no CD73 inhibition). The mean concentrations of endogenous adenosine in MIX 1 samples were 20 nmol/L as compared to 10 nmol/L observed in samples collected into STOP. Much lower mean concentrations, 2 nmol/L and 3 nmol/L, were found in samples collected into MIX 2 (no AK and ADA inhibition) and NO STOP respectively (Fig 2D).

### Performance of the defined STOP solution

**Fig 3:**
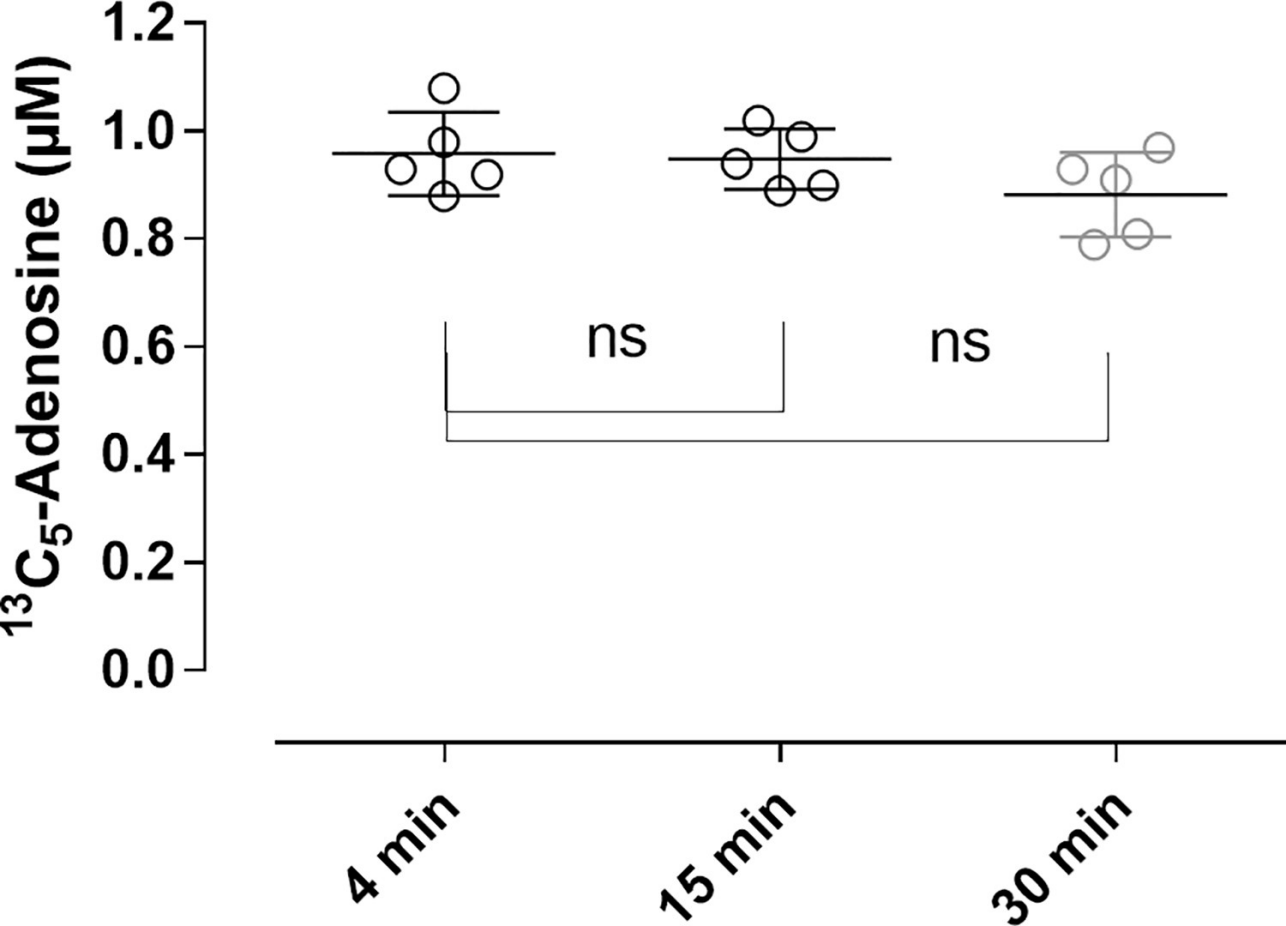
Stability over time of exogenous ^13^C_5_-adenosine in blood collected into STOP solution. Venous blood from healthy donors was collected into blood collection tubes pre-filled with STOP solution containing a final blood concentration of 1μmol/L 13C5-adenosine. Plasma was prepared at 4, 15 and 30 min after blood collection. Data expressed as means ±SD, n = 5.

The defined STOP solution inhibited adenosine clearance in blood with >97% and >92% ^13^C_5_-adenosine conserved in plasma samples prepared after 15 and 30 minutes compared to plasma samples prepared after 4 minutes (Fig 3). The reduction in ^13^C_5_-adenosine at 15 and 30 minutes was not statistically significant relative 4 minutes. Based on comparisons of concentrations in blood-free reference samples with PBS mixed into the STOP and tracer solutions the measured concentrations of ^13^C_5_-adenosine after 4 minutes support >95% conservation.

**Fig 4:**
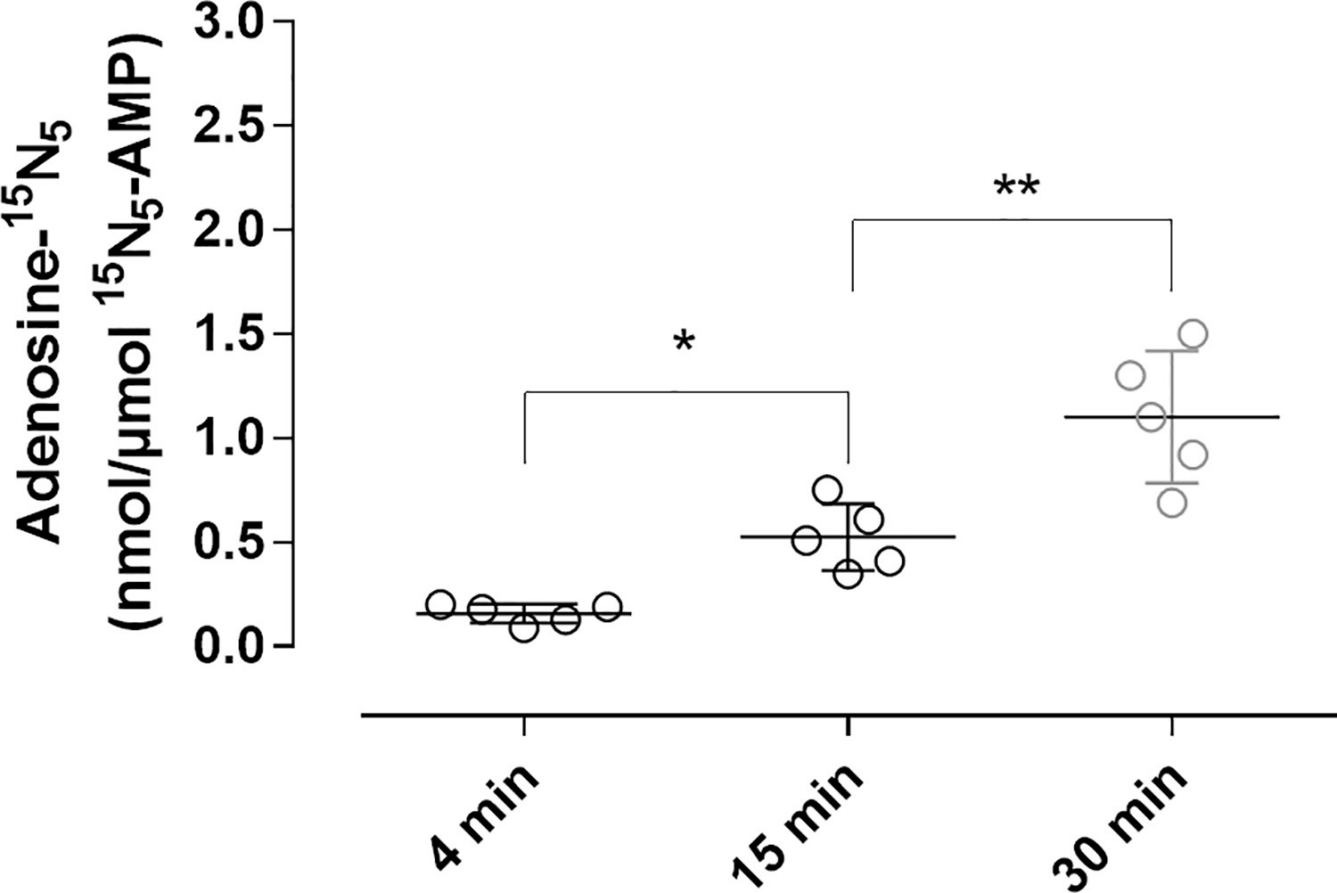
^15^N_5_-adenosine formation over time by degradation of exogenous ^15^N_5_-AMP in blood collected into STOP solution. Venous blood from healthy donors was collected into blood collection tubes pre-filled with the STOP solution containing a final blood concentration of 10 μmol/L ^15^N_5_-AMP. Plasma was prepared at 4, 15 and 30 min after blood collection. Data expressed as means ±SD, n = 5.

The defined STOP solution also effectively inhibited adenosine formation from AMP in blood (Fig 4). In plasma samples prepared after 30 min 1 nmol/L ^15^N_5_-adenosine per μmol/L ^15^N_5_-AMP had been hydrolysed corresponding to 99.90% inhibition of AMP conversion to adenosine. In plasma samples prepared after 4 and 15 min the AMP to adenosine conversion was inhibited by 99.99% and 99.95% respectively.

**Fig 5:**
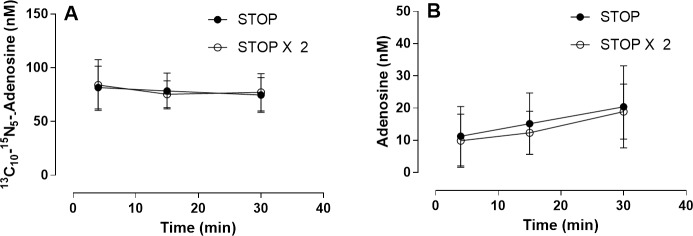
**Comparing STOP and STOPx2 performance in conservation of exogenous** ^**13**^**C**_**10**_**-**^**15**^**N**_**5**_**-adenosine (A) and measured endogenous adenosine concentrations (B)** Venous blood was collected into blood collection tubes pre-filled with STOP and STOPx2 containing 100 nmol/L ^13^C_10_-^15^N_5_-adenosine final blood concentrations. Plasma was prepared at 4, 15 and 30 min after blood collection. Data expressed as means ± SD, n = 5.

The performance of the STOP solution was similar to the STOPx2 solution as both the stability of exogenous ^13^C_10_-^15^N_5_-adenosine (100 nmol/L) and endogenous adenosine was close to identical (Fig 5), confirming adequate composition of the STOP solution. The mean endogenous adenosine concentration in plasma samples prepared at 4, 15 and 30 min after blood collection into STOP solution was 11, 14 and 19 nmol/L, respectively.

### Accurate measurement of endogenous adenosine in human blood

**Fig 6:**
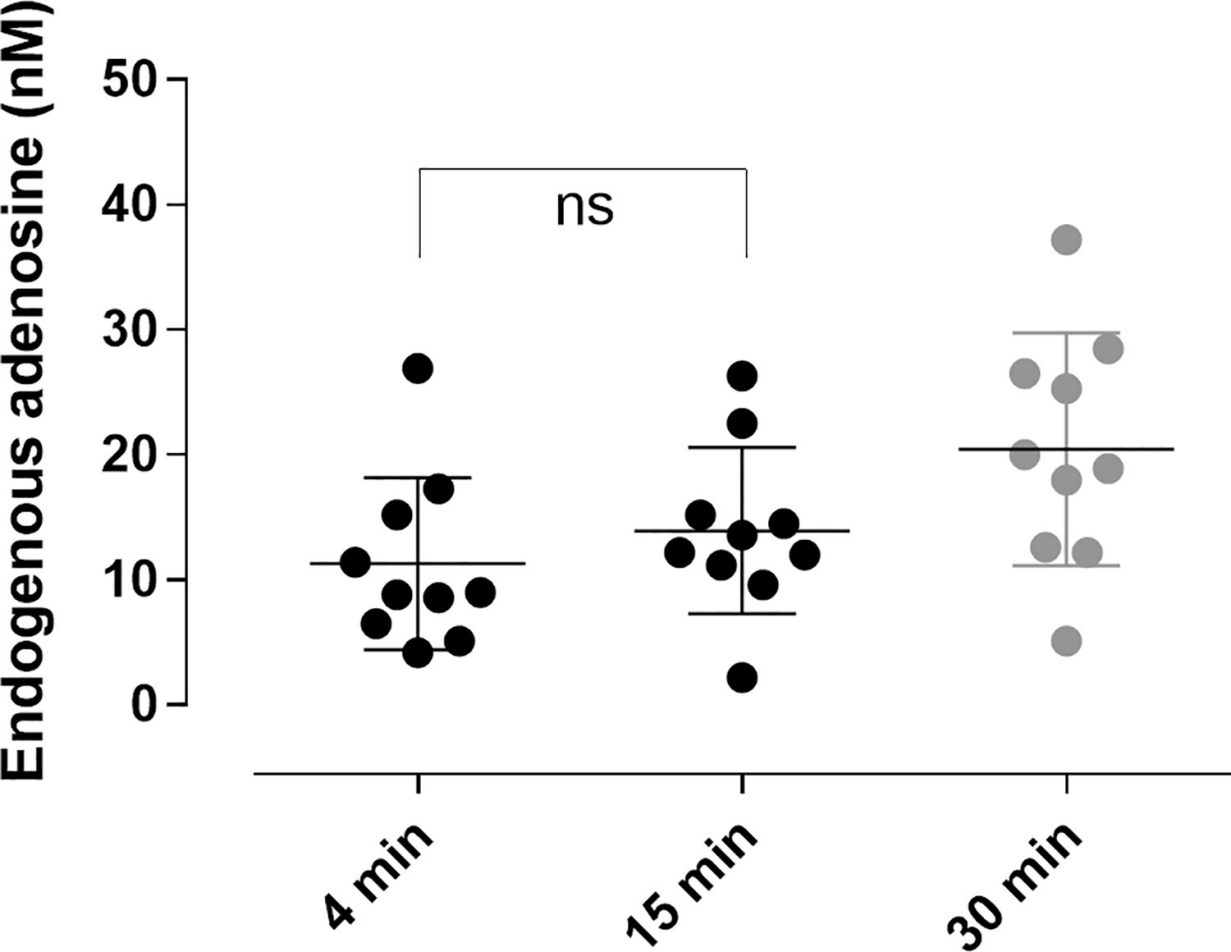
Endogenous adenosine in human blood plasma. Venous blood was collected into blood collection tubes pre-filled with STOP solution. Plasma was prepared at 4, 15 and 30 min after blood collection. Data expressed as means ±SD, n = 10.

Using the defined STOP solution, the mean endogenous plasma adenosine concentrations were 11 ± 7 nmol/L, 13 ± 7 nmol/L, and 20 ± 8 nmol/L in plasma prepared 4, 15 and 30 min post blood collection (Fig 6). No degradation was observed for adenosine in plasma stored 1 month at -20°C or -80°C or kept at room temperature for 2 hours prior to analysis or in plasma exposed to repeated freeze-thaw cycles (S1 Table).

### Ticagrelor effects on adenosine conservation

**Fig 7:**
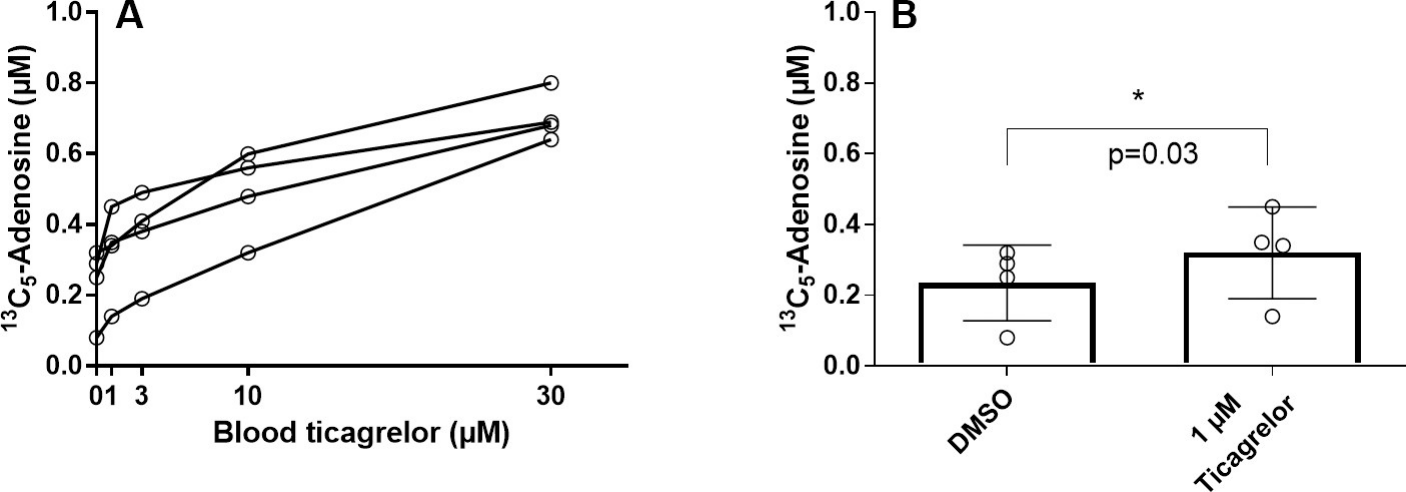
Ticagrelor effects on exogenous ^13^C_5_-adenosine clearance in blood. Venous blood was pre-incubated *in vitro* with ticagrelor for one h. Pre-incubated blood was spiked with a final blood concentration of 1 μmol/L ^13^C_5_-adenosine and mixed by gently inverting the tubes eight times. After 1 min, blood was transferred into the STOP solution and plasma was directly prepared. Data expressed as means ± SD, n = 4. p value by one-tailed paired t-test.

Ticagrelor concentration-dependently inhibited ^13^C_5_-adenosine clearance (Fig 7A). The effect was significant already at 1 μmol/L ticagrelor (Fig 7B).

### Adenosine concentrations after filtration or perchloric acid (PCA) extraction

Endogenous adenosine concentrations in PCA-treated samples were identical to results obtained by the validated filtration method. The time from PCA addition to neutralization (5 min or 12 hours) had no effect on the adenosine concentrations. Addition of AMP to the samples prior to filtration or PCA extraction did not increase adenosine concentrations. The stability of exogenous ^13^C_10_-^15^N_5_ adenosine was close to 100% and did not differ between protocols as 99 ± 5.6% (PCA, 5 min + neutralization), 101 ± 3.8% (PCA, 12 hours + neutralization) and 100 ± 4.5% (filtration) was recovered.

## Discussion

The primary outcome from the present study is the defined blood sampling protocol, focusing on of the composition of the STOP solution used to conserve the *in vivo* adenosine concentrations. Together with a validated analytical method for plasma adenosine, this enables accurate measurement of *in vivo* extracellular adenosine concentrations in human blood samples.

Ultra-performance liquid chromatography coupled to tandem mass spectrometry (UPLC-tandem-MS) has been shown to offer superior performance over other detection methods for adenosine in terms of accuracy, sensitivity, specificity, sample volume requirements and speed [12–14]. Finally, UPLC-tandem-MS enables the use of more relevant internal standards as opposed to other techniques. Stable isotope labelled ^13^C_5_-adenosine serves as the ideal internal standard for adenosine assessment by UPLC-tandem-MS UPLC-tandem-MS is therefore the method of choice for accurate assessment of plasma adenosine.

The method described here with a LLOQ of 2 nmol/L was validated according to the European Medicines Agency guidelines. The method delivered high precision and accuracy as well as robustness and ruggedness as confirmed by the validation data provided in S1 Table.

Previous publications have reported diverging human plasma adenosine concentrations, 10–900 nmol/L, when using a range of different STOP solutions [5, 15–19]. We set out to define the optimal STOP solution by using stable isotopes of adenosine and AMP in combination with mass spectrometry. With these tools the relevance of the different contributors to adenosine formation and clearance was visualized with high sensitivity and precision. This enabled definition of the optimal concentrations of inhibitors of the enzymes and transporters involved in the STOP solution.

In the absence of any inhibition (NO STOP) or in the absence of ADA and AK inhibition (MIX 2) no trace of added adenosine isotope could be detected in plasma prepared 5 min after blood collection highlighting the absolute need for inhibition of these enzymes responsible for adenosine clearance. In contrast when collected into STOP solution, similar quantities of adenosine remained in plasma when prepared 15min after blood collection compared to the plasma sample prepared 4 minutes after blood collection and this difference was not statistically significant. Based on comparisons of concentrations in blood-free reference samples with PBS mixed into the STOP and tracer solutions >95% of added adenosine remains after 4 minutes.

Equally important as inhibiting the adenosine clearance is to inhibit adenosine formation via 5-NT. Using only inhibitors of adenosine clearance will inevitably result in over-estimation of adenosine, as previously demonstrated [5]. This is also evident from our data where a large amount of adenosine is formed from AMP when blood is collected into a solution lacking 5-NT inhibition but containing ADA and AK inhibition (MIX 1). Under these conditions AMP is rapidly degraded to adenosine. The formed adenosine is protected by the ADA and AK enzyme inhibitors and only minor amounts of hypoxanthine are formed. On the other hand, when blood is collected into a solution containing 5-NT inhibition but lacking ADA and AK enzyme inhibitors, only minute amounts of adenosine generated from AMP could be detected as the adenosine formed is further degraded to hypoxanthine. Extrapolation from the reported 1–2 μmol/L AMP in the circulation [20] and the reported rate of AMP hydrolysis of about 1 μmol/L per minute [21] predicts that a maximum of 15000 nmol/L adenosine could be formed after 15 minutes (assuming complete conversion from AMP and no adenosine clearance). The different concentration of 5-NT inhibition used in different STOP solutions is thus most likely a major reason for the diverging adenosine plasma data reported. Our stable isotope data suggest 99.95% inhibition of adenosine formation from AMP. This level of inhibition is also supported by the 2 nmol/L observed mean increase in endogenous adenosine when preparing plasma from the blood:STOP mix at 4 vs 15 minutes post collection.

Using the defined blood sampling protocol, STOP solution and adenosine analytical method the mean plasma adenosine concentration in venous blood collected from healthy subjects was 13 ± 7 nmol/L. These results are in agreement with previous observations by Ramakers et al [5] reporting 12 ± 2 nmol/L plasma adenosine in venous blood from healthy donors. Ramakers used a custom-made syringe allowing immediate mixing of their inhibitor solution with the blood. The agreement with our data indicates that our simple sampling procedure provides efficient mixing comparable to that obtained with the custom-made syringe used by Ramakers. As a sensitive test to confirm that the composition of the final STOP solution was optimal we compared its performance with a solution, STOPx2, containing double concentrations of all the inhibitors included. The performance of STOPx2 and STOP was very similar supporting the suitability of the final composition of STOP.

As well as the composition of the STOP solutions and the time for the collected blood to mix with the STOP solution there are other potential sources for non-accurate estimation of in vivo adenosine concentrations, including protein removal. We used filtration in our protocol but others use perchloric acid protein precipitation [15–17]. The selection of methodology may be important as potential acid-catalyzed dephosphorylation of AMP can induce false high plasma adenosine concentrations. However, AMP stability and adenosine stability was demonstrated when comparing both methods.

We recommend that plasma is prepared within 15 minutes of blood collection in STOP solution. However, the time in plasma may also be important. Even if adenosine clearance is mainly restricted to the intracellular space, some ADA activity is likely present in plasma. Our validation data support that our STOP solution prevents adenosine clearance in plasma over at least 2 hours at room temperature and at least during 1 month’s storage in freezers and during repeated freeze-thaw cycles.

Finally, we used the STOP solution and adenosine analytical method to explore the ability of ticagrelor, a known ENT1 inhibitor, to conserve exogenous adenosine added to whole blood in vitro. Ticagrelor concentration-dependently conserved the added adenosine and the effect was significant at 1 μmol/L ticagrelor exposure and above, confirming prior data using a different methodology [9]. This effect is clinically relevant as the mean maximal and minimal plasma concentrations of ticagrelor in patients following 4 weeks of treatment (90 mg bid) has been reported to 1.5 μmol/L (770 ng/mL) and 0.4 μmol/L (227 ng/mL) [22]. Indeed, ticagrelor has been shown to augment adenosine-induced coronary blood flow after clinical exposure both in healthy subjects and ACS patients [23–24]. Ticagrelor has also been described to increase adenosine plasma concentrations in patients and healthy subjects in many [16–17, 25] but not all studies [19, 26–27]. The differences may in part be due to the differences in methodology especially the composition of the STOP solution discussed here. Similar plasma adenosine concentrations, as we report here for healthy subjects, were observed in patients in the recently reported HI-TECH clinical trial where the here presented STOP solution and validated LC-MS method was used for blood collection and adenosine analysis [27]. Another critical aspect that warrants further exploration is accurate measurement of local adenosine concentrations at sites of tissue injury and hypoxia since higher concentrations than those circulating in plasma can be expected. Drugs impacting adenosine clearance such as ticagrelor would be expected to have a greater impact on local concentrations as the circulating concentrations are very low and therefore hard to impact. A local impact is unlikely to be mirrored in the plasma due to dilution and time of circulation. So far, the impact of ticagrelor on local adenosine concentrations at sites of ischemia has only been studied in a rat model of ischemia reperfusion injury. In this model ticagrelor increased heart adenosine concentrations with an additive effect in combination with rosuvastatin [28–30].

## Conclusion

We report here an optimized blood collection protocol and a validated UPLC-tandem-MS method for accurate measurement of in vivo circulating adenosine concentrations in human blood suitable for clinical trials.

## Supporting information

S1 Table (DOCX)
