# Supplementary material for: Accurate measurement of endogenous adenosine in human blood
Source: PLoS One. 2018 Oct 25;13(10):e0205707. doi: 10.1371/journal.pone.0205707 (PMC6201894; doi:10.1371/journal.pone.0205707)
Supplement: S1 Table — (DOCX) [file pone.0205707.s001.docx]

**S1 Table**

**Accurate measurement of endogenous adenosine in human blood**

Lars Löfgren^1^, Susanne Pehrsson^1^, Gunnar Hägglund^2^, Henrik Tjellström^2^ and Sven Nylander^1^

^1^ Cardiovascular, Renal and Metabolism, IMED Biotech Unit, AstraZeneca, Pepparedsleden 1, 431 83 Mölndal, Sweden

^2^ Q&Q Labs AB, Pepparedsleden 1, 431 53 Mölndal, Sweden

Correspondence to:

Dr Lars Löfgren

E-mail: [lars.lofgren@astrazeneca.com](mailto:lars.lofgren@astrazeneca.com)

Phone: (+46) 31 7762381

**Abstract**

A high sensitive ultra performance liquid chromatography-tandem-mass spectrometry (UPLC-tandem-MS) analytical method for accurate measurement of plasma adenosine was developed and validated with a lower limit of quantification of 2 nmol/L (S1 Table). The here described analytical method for endogenous adenosine assessment in human plasma was validated according to GCP (good clinical practice) in accordance with a validation plan based on the EMEA Guidelines.

Parameters evaluated and validated were selectivity, matrix effects, accuracy, calibration curves, instrument repeatability, carry-over, dilution integrity, lower limit of quantitation (LLOQ) and sample stability. The performed validation tests and results are summarized in S1 Table.

The method demonstrated plasma adenosine stability during sample processing and analytical method performance relevant to human blood samples. In conclusion, we report an optimized sampling protocol and a validated analytical method for accurate measurement of *in vivo* circulating adenosine levels in human blood suitable for clinical trials.

**S1 Table. Summary of method validation for endogenous adenosine in human plasma^1)^**

| **Validation Parameter** | **Acceptance Criteria** | **Result** |
| --- | --- | --- |
| Selectivity | The analyte signal from the internal standard should be < 5% at LLOQ | Accepted. There was no adenosine signal from the ^13^C_5_-adenosine internal standard in matrix-free samples and no elevation of the endogenous plasma adenosine signal upon addition of internal standard. |
| Matrix Effects | The CV of the internal standard normalised matrix factor should be <15%. | Accepted. CV < 1 % was observed. Blood from 6 donors were spiked at 50% and 75% of highest calibration level. The internal standard compensates for matrix effects. |
| Accuracy and precision Within-Run | Relative standard deviation (RSD) < 15% above LOQ and <20% at LLOQ (2 nmol/L) | Accepted. RSD <15% at all four QC levels including LLOQ. n=5 at each QC level. Accuracy was within 85-115% (80-120% at LLOQ). |
| Accuracy and precision Between-Run | RSD < 15% above LLOQ and <20% at LLOQ (2nmol/L) | Accepted. RSD <15% at all four QC levels including LOQ. n=15 (3 days x 5 samples). Accuracy was within 85-115% (80-120% at LLOQ). |
| Calibration curves | Correlation coefficient R^2^ >0.995. Back-calculated value within 85-115% (80-120% at LLOQ). | Accepted. 6-point (n=3) calibration curve (2-500 nmol/L) run before and after QC samples. Correlation coefficient was >0.995. Back-calculated values were within 85-115% (80-120% at LLOQ). |
| Repeatability | <15 % RSD | Accepted. RSD was < 1% and no drift was observed for 10 injections of QC sample. |
| Carry-Over | < 20% of LLOQ and <5% of Internal standard | Accepted. No signal of analyte or internal standard detected in blank samples analysed after a matrix sample |
| Dilution Integrity | 85 – 115% accuracy with CV <15% | Accepted. Plasma spiked at 5 times ULOQ injected at 10% (0.8 µL) of normal volume resulted in 85 – 115% accuracy with CV <15% |
| LLOQ | Signal–to-Noice (S/N) > 5 | Accepted. LLOQ 2 nmol/L defined with S/N >10 observed |
| Sample Stability | < 10% degradation after 2 h at room temperature, 3 freeze-thaw cycles, and long-term storage for 1 months at -20C and -80C respectively. < 10% degradation during 24 h storage of sample filtrate in auto-sampler | Accepted. There was no significant degradation observed in any of the stability tests and no change of concentrations in the sample filtrates stored in the auto-sampler |

1. Plasma prepared from blood collected in the STOP solution
